# Integrating refugees into national health systems amid political and economic constraints in the EMR: Approaches from Lebanon and Jordan

**DOI:** 10.7189/jogh.12.03008

**Published:** 2022-03-28

**Authors:** Shadi Saleh, Sarah Ibrahim, Jasmin Lilian Diab, Mona Osman

**Affiliations:** 1 Global Health Institute, American University of Beirut, Beirut, Lebanon; 2 Department of Health Management and Policy, Faculty of Health Sciences, American University of Beirut, Beirut, Lebanon; 3 Department of Social Sciences, School of Arts and Sciences, Lebanese American University, Beirut, Lebanon; 4 Institute for Migration Studies, School of Arts and Sciences, Lebanese American University, Beirut, Lebanon; 5 Department of Family Medicine, Faculty of Medicine, American University of Beirut, Beirut, Lebanon

## REFUGEE HEALTH IN THE EMR

Refugees are often among the most vulnerable of society’s members. The Office of the United Nations High Commissioner for Refugees [1] reports that as of 2021, globally, the number of forcibly displaced population is 80 million – the highest level of human displacement ever, and includes 26.3 million refugees [1]. There are also 10 million stateless people who lack a nationality and access to basic rights such as education, health care, employment and freedom of movement [1]. The Eastern Mediterranean Region (EMR) is an epicenter of refugees and the displaced population crisis. Currently, the region hosts almost two-thirds (16.7 million) of the total number of refugees worldwide (26.3 million) and 33% (1 million) of the world’s asylum seekers (3.1 million) [1].

Refugees and displaced populations have limited access to health care services, including health promotion, disease prevention, treatment and care, as well as financial protection which is concerning [2]. This vulnerable population faces a considerable public health challenge, including but not limited to, poor mental health status, poor reproductive, maternal and child health, an increase in non-communicable diseases (NCD), communicable diseases, as well as long-lasting casualties and injuries [2]. The public health consequences of refugee populations emerging in the EMR are profound and persistent, affecting not only the vulnerable populations but also the host community and health security of the entire region [2]. For instance, the EMR has one of the highest prevalence of NCDs globally, with over 100 million people living with hypertension, 50 million people with diabetes mellitus and 1.35 million with cancer in 2021 [3]. The influx of refugees in the EMR further amplified this burden with an estimated prevalence of hypertension in more than 50% of Syrian refugees residing in Lebanon, reaching 76% in a number of studies [4-7]. As for mental health, a study by Global Health Institute in Lebanon revealed that an estimated one in four Syrian refugees shows moderate to severe depressive symptoms [8]. Additionally, child health among the refugee population is compromised with increased prevalence of acute respiratory disease, acute malnutrition and micronutrient deficiencies due to inappropriate infant and young child feeding practices, and diarrhea due to limited access to safe water [2]. This is compounded by the fact that refugee women have limited access to sexual and reproductive health care services [9,10]. In fact, many refugee women do not receive antenatal care or face difficulties receiving the service due to payment barriers, lack of access to a gynecologist, lack of access to information or fear [11].

## THE IMPACT OF COVID-19 ON REFUGEES IN THE EMR

With the pre-existing fragile situation of refugees, the COVID-19 pandemic outbreak exacerbated the precarious living conditions and public health challenges among this population [12]. Refugees are at an increased risk of contracting the COVID-19 virus as they typically live in overcrowded settlements with the absence of basic amenities such as clean running water, soap, and basic sanitation [13]. Moreover, refugees are affected by income loss and health care insecurity which further worsen their health status [13].

According to the 2020 vulnerability assessment of Syrian refugees report in Lebanon (VASyR) following the impact of COVID-19, an increase in the prevalence of households living in extreme poverty was evident increasing from 55% in 2019 to 89% in 2020 [14]. In the pandemic, the VASyR report revealed an increase in the cost of health care (out of pocket expenditure) which remained overwhelmingly the greatest barrier to receiving primary health care, including drugs, doctors’ fees, and transportation costs [14]. However, since the beginning of the Covid-19 outbreak, the UNHCR has been committed to collective efforts to prevent and contain transmission of the virus [15]. By providing prevention services through distributing hygiene materials, and containing transmission, through enabling quarantine of refugees living in overcrowded settlements in 13 isolation facilities, the majority (>90%) of COVID-19 cases were detected among refugees in urban settings with no major outbreaks in the vulnerable informal settlements [16].

Similarly, in Jordan, the rapid needs assessment report (2020) following the COVID-19 outbreak reported that 90% of the refugees do not have enough money to cover their basic needs during the lockdown measures [17]. In addition, approximately 17% of the refugees in Jordan had a household member with a chronic illness. Travel restrictions, inadequate cash, and closure of facilities were the main challenges to access health care services by the refugees in Jordan [17]. In parallel to Lebanon, UNHCR responded with measures to prevent spread and contain the virus within the refugee population inside and outside of camps [18]. Thus, 3% of the total refugees in the camp population tested positive since the onset of the pandemic, compared to 6.7% of the total Jordanian population [18]. However, there may be a discrepancy in the availability and access to testing, which may have impacted the abovementioned numbers. In summary, the underlying vulnerability and poor income/employment among Syrian refugees have further deteriorated due to the pandemic [19].

The current pandemic highlights how a population without access to essential services creates a risk for the concerned individuals along with the entire population in general [2]. According to the UN high commissioner for refugees, Filippo Grandi, “if ever we needed reminding that we live in an interconnected world, the novel coronavirus has brought that home” [20]. Refugees face challenges to attain essential health care services, thus integrating them into the national health system is pivotal.

## OVERVIEW OF REFUGEES AND HEALTH CARE ACCESS AND EQUITY

Several international organizations play an important role in integrating refugees into the national health system. The UNHCR works with partner organizations and ministries of health to ensure refugees receive the health support and medical treatment needed in emergencies and in stabilized and protracted situations [21]. The United Nations Relief and Works Agency (UNRWA) works closely with Palestine refugees, and Palestine refugees from Syria, to provide health care services and support [22]. Additionally, the World Health Organization [11] provides global leadership and guidance in public health matters within the United Nations. The WHO has a primary responsibility to ensure health for all and universal health coverage within the 2019-2030 Agenda for Sustainable Development and its associated Goals, by leaving no one behind [23]. Implementing the global action plan requires strong coordinated work and management of refugee health at all levels, and in collaboration with UNRWA, WHO and UNHCR, as well as other international organizations and stakeholders.

Achieving Universal Health Coverage (UHC) in Lebanon remains a challenge on various levels. The health care system in Lebanon is based on a public-private partnership between multiple sources of funding that is highly fragmented [24]. Around 238 primary health care centers (PHCC) are available and supported by the Ministry of Public Health (MoPH). Of the 238 PHCC, 68% are from civil society/NGO, 13% from municipalities, 8% are from mixed municipalities and NGO partnerships, 10% governmental and 1% are from academic institutions [25]. Nearly half of the population is financially covered by the National Social Security Fund (NSSF), government funds, or private insurance [24]. In fact, a total of six public funds are available in Lebanon. The MoPH financially covers the uninsured as the ‘insurer of last resort’ [26]. Despite multiple insurers, 53% of the Lebanese population rely on the ‘insurer of last resort’ from the MoPH. Additionally, Lebanon has a high out of pocket health expenditure, exposing vulnerable households to financial risk [26]. Despite the wide availability of health care services to all people residing in Lebanon (including refugees), unequal access to health care remains a barrier due to geographic locations.

**Figure:**
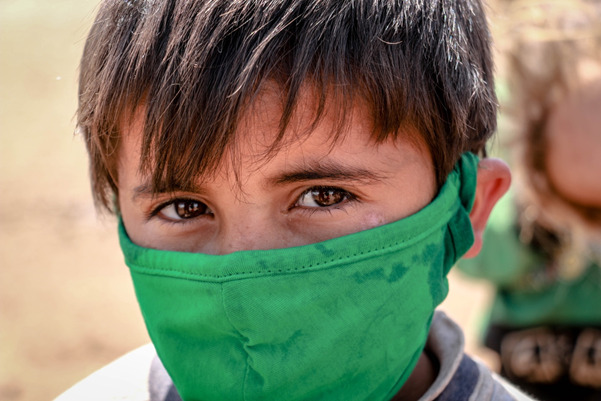
Photo: From https://www.pexels.com/photo/a-young-boy-wearing-a-green-face-mask-7385963/.

With a total population of six million, Lebanon has the highest number of refugees per capita in the world, whereby Syrian refugees make up 1.5 million of the total population [27]. Most of the refugees in Lebanon reside in low-resource areas, making it difficult to access the available health care services [25]. The provision of health care for Syrian refugees is primarily through a collaborative effort between the Lebanese Ministry of Public Health (MoPH), UNHCR, and humanitarian agencies (NGO’s) [25]. Syrian refugees have access to over 200 primary health care facilities with free medication and vaccines [25]. In fact, according to the UNHCR, health care provision for these Syrian refugees has been resilient and has improved since the high influx of refugees following the Syrian conflict in 2011. The improvement in health care was depicted by an increase in the availability of health care centers along with the type of health care including specialized treatment such as psychological aid [25]. Yet, economic factors, such as the cost of consultation and laboratory tests remain a significant barrier for many refugees despite the help provided by the NGO’s. Additionally, access to secondary and tertiary care remains a growing challenge for the Syrian refugees in Lebanon.

Currently, there are no formal refugee camps established in Lebanon for Syrian refugees as per government policy [28]. Thus, Syrian refugees are scattered around the country in cities, villages, or informal tented settlements (IS’s) [28]. Mobile Medical Unites (MMU), targeting the most vulnerable individuals in the ISs are used to provide uniformed basic primary health care service by UNHCR [29]. Syrian refugees pay a subsidized fee of US$2-3.33 per consultation at contracted PHCCs and 85% of diagnostic costs are covered for Syrian refugees below 5 years of age, above 60 years of age, disabled, pregnant, or lactating women [30]. Refugees requiring secondary or tertiary care require referrals by a UNHCR contracted PHCC. Extremely vulnerable refugees have 100% of hospital care costs covered by UNHCR, while the remaining get 75% coverage in the case of life-threatening events [30]. Additional barriers faced by Syrian refugees include lack of clarity for eligibility criteria, perceived discrimination, affordability, and transportation [30].

Lebanon currently also approximately half a million Palestine refugees who are registered with the UNRWA. Close to half of these reside in camps with complicated health problems emerging from the deteriorating living conditions, low wages, high poverty and deprivation [31,32]. Palestinian refugees in Lebanon are ineligible for the State’s health care services. They can only access health care through private sectors, which has high fees, or by international organizations such as UNRWA [33]. UNRWA operates 28 primary health care facilities, providing both preventive and curative care [34]. The UNRWA formed an agreement with Palestine Red Crescent Society (PRCS) to provide equity for Palestinian refugees in accessing secondary health care services [33]. The UNRWA also works on decreasing the financial burden borne by the refugees by providing services such as family planning, preconception care, antenatal and postnatal follow-up, infant care, school health, oral health, outpatient consultations, diagnostics, laboratory services, vaccination, among others [35]. In other words, UNRWA is playing the role of the Ministry of Health to provide Palestine refugees with health care essential services.

With the aforementioned precarious health-related conditions that refugees are facing, efforts to integrate refugees in the national health systems are important and have achieved some progress, however, there are many ongoing challenges and significant support will be required to continue this effort. The ability of the existing health system to provide UHC, even for its citizens, is challenging. One challenge is the unclear policy framework for health care provision from the government, another is the overwhelming predominance of unregulated private sectors in the financing and provision of health care. In fact, secondary and tertiary care provision is almost exclusively given by the private sectors. Additionally, the Lebanese health care system is fragmented by target groups where each social group has access to different services and a different level of social protection [36]. This type of a fragmented health system has been proven to be inequitable and inefficient [37]. Another key challenge is the out of pocket (OOP) expenditure on health, which in Lebanon, has already reached 33.2%, which WHO has deemed catastrophic [37]. Furthermore, Lebanon’s political and economic instability, along with the COVID-19 pandemic and the August 4 2020 explosion, further amplifies the burden on achieving UHC and in seeking medical care. Due to the aforementioned overlapping crises, seeking medical care became extremely difficult. Additionally, the ability to lead a healthy lifestyle has become increasingly difficult with a surge in the price of food and basic necessities aggravating chronic diseases [36]. Lastly, Lebanon is also witnessing a shortage in drug supply making it difficult for everyone to attain their daily prescribed medications [36]. Despite all of the above challenges, it remains imperative to integrate refugees into the health system. By achieving this, inequities and inefficiencies between the public, private and humanitarian sectors will be mitigated [38].

The health sector in Jordan consists of a multi-segmented public and private program [39]. The two major public programs that deliver health care services are the Jordan Ministry of Health (JMoH) for civil health and the Royal Medical Services (RMS) for the military program. Other public programs that provide health care services are university-based programs. In addition to the public services, NGOs such as UNRWA and UNHCR provide services to Palestinian and Syrian refugees in Jordan (similar to Lebanon). Owing to economic development and political stability, Jordan has built one of the strongest health sectors in the EMR [40]. Public primary health care services are widely available and distributed across the country. With its resilient health sector, Jordan faces fewer challenges in providing health care services and achieving UHC. The current segmented health care system in Jordan remains the main obstacle towards achieving UHC [39]. Another obstacle is high out of pocket expenditure for the uninsured [39]. Other challenges faced by the health sector include an aging population, the growing burden of chronic diseases, and significant pressure on public services resulting from the high influx of refugees [39]. Lastly, although public primary care is widely available across the country, specialized care is rather concentrated in urban areas with a very weak referral system [40].

As of 2021, Jordan hosts one of the largest number of refugees in the EMR, with approximately 2 million registered Palestinian refugees (with UNRWA), and 665 000 Syrian refugees registered with the UNHCR [41]. Unlike Lebanon, Palestinian refugees in Jordan are entitled to full Jordanian citizenship, which includes health care and participation in political and economic life. Palestinian refugees reside mainly in ten official and three unofficial camps where UNRWA provides health and other services [42]. Similar to Lebanon, UNRWA provides the health care services for nearly 56% of the registered Palestinian refugees in the country, providing over 1.9 million general consultations and over 67 000 dental screening each year as well as family planning services and NCD support, among others [43].

As for the Syrian refugees in Jordan, five camps were formed to accommodate the displaced individuals with Zaatari camp being the largest, hosting approximately 12% (80,000) of such refugees. The camp consists of 12 distinct districts, 58 community centers, 32 schools and 8 medical centers which are under the joint administration of the UNHCR and the Syrian Refugee Affairs Directorate (SRAD) where refugees have free access to health care services and resources [44,45]. However, the majority (84%) of the registered Syrian refugees live in urban areas outside of refugee camps [44,45]. Similar to Lebanon, the JMoH provides full access to health care services for Syrian refugees residing outside of refugee camps. In fact, Syrian refugees in Jordan are integrated in the health system and can use the public services as Jordanians [44,45]. Despite the access granted to the public health care system and the UNHCR benefits, seeking health care services remains a burden on the Syrian refugees as most do not work or have a high out of pocket expenditure [44,45].

Despite the enormous efforts by the humanitarian sectors, such as UNHCR and UNRWA to provide equal health care services to refugees, a UHC plan needs to be implemented. The humanitarian agencies, such as the UNHCR, are experiencing a shortage of funding which may have a great impact on the refugee population [46]. Additionally, in line with the above-mentioned information, the humanitarian agencies are not capable of achieving full health coverage to all refugees alone. It is therefore crucial to implement reformations in order to achieve the 2030 UHC goal.

## CONCLUDING REMARKS

The ability of existing health systems to provide health care is impacted by many factors including the loss of physical, human and institutional infrastructure; insecurity and instability; protracted emergencies and forcible displacement; ongoing conflicts with no foreseeable resolution in place; and uncertainty in the areas of economic burdens and political will. To meet the shared sustainable development goals (SDG)’s on health, each and every person’s right to health needs to be upheld, respected, protected and fulfilled. UHC and the human right to health go hand in hand as mutually inclusive realities that governments must be held accountable for. Moreover, governments are not only responsible to their populations, but also to individuals present on their territory in compliance with their international humanitarian and legal obligations. In efforts to ensure the provision of UHC in compliance with the right to health, governments must reassess their policies, legal frameworks, health systems and practices in order to evaluate whether or not they uphold the rights of refugees.

The universality of the SDGs aims to ensure that everyone – including refugees– have access to a minimum standard of health care services. The approach undertaken by the health care systems in Lebanon and Jordan at present are unable to include refugees in the access to essential services without the involvement of international humanitarian organizations, mostly due to the fact that both countries, similar to others in the region, are themselves fragile when it comes to context and capacity. In Lebanon, achieving UHC remains a main goal of the National Health Strategy. However, its feasibility is questionable due to economic and political obstacles. To begin with, financing sources in Lebanon are heavily skewed towards domestic private expenditure and OOP spending. In 2017, OOP spending accounted for 33% of funding for health service in the Lebanese health system. Additionally, Lebanon’s high health GDP of approximately 8%-10% (which is comparable to many European countries) does not reflect better care, services, or health for the Lebanese or refugee population as the majority of the expenditure is concentrated in high-tech-high-cost interventions used by a small number of patients, with little to no focus on basic primary and preventive health care services. Furthermore, with Lebanon facing one of its greatest economic crises, funding for achieving UHC, more so integrating refugees into the national health system, is difficult and may only be possible through the help of humanitarian organizations.

Integrating refugees in the national health system now, at a time where Lebanon is facing a great political instability crisis, could lead to greater challenges. This is because, integrating refugees in the national health system would infer that the government is accepting their permanent presence in Lebanon. This is particularly true for Palestinian refugees, whose presence in Lebanon has lasted for decades amid historical and political sensitivities. However, having a parallel functioning system (with UNRWA and UNHCR) is not efficient or sustainable in the long run; and reforms are necessary. With that being said, integrating refugees into the national health system may be difficult to achieve in the short run; however, it is important to move closer towards UHC and gradually integrate refugees into the health system while factoring in the political and economic complexities of the country. Ultimately, the goal and hope is for refugees to settle back in their respective countries.

On the other hand, Jordan’s health care system has been shown to be one of the strongest health systems after the COVID-19 pandemic. However, with its growing national population and influx of refugees, Jordan struggles to ensure that everyone, including refugees, can access quality health care. Jordan faces a dual challenge of extending the overburdened health services, to meet the increased demand, while continuing to strengthen the quality of services. Additionally, the limited financing in the health care system is also a great factor that delays the progress towards UHC in Jordan. With that being said, it is pivotal for Jordan to expand their health care infrastructure and workforce in order to accommodate more people. Jordan must aim at improving the accessibility of public health services, reaching the poorest, and most vulnerable. With their continuous efforts in achieving UHC, Jordan faces challenges pertaining to the financial and material aspect in including everyone in the national health care system.

The main obstacle that both Jordan and Lebanon currently face is the lack of sustainable resources, and subsequently/in parallel the political will, to officially integrate refugees in the national health system. It is pivotal that the fate of refugees be detached from protracted conflicts and the political sensitivities surrounding them. Though Jordan has initiated steps in incorporating refugees into the society at all levels by allocating a department in the government, Jordan Response Plan (JRP), aimed at outlining the rights of refugees, human rights considerations remain inadequate in addressing systemic and intersectional issues in the areas of access to adequate standards of aid and protection. Both countries signed the Universal Health Coverage 2030 Global Compact, demonstrating the high level of commitment to take action and progress towards UHC, not integrating refugees in the health system will continue to be an obstacle to achieving that.
